# Applications and Efficacy of Iron Oxide Nanoparticles in the Treatment of Brain Tumors

**DOI:** 10.3390/pharmaceutics17040499

**Published:** 2025-04-09

**Authors:** London Varalli, Reed Berlet, EC Abenojar, John McDaid, David A. Gascoigne, Julian Bailes, Daniil P. Aksenov

**Affiliations:** 1 Department of Radiology, Endeavor Health, Evanston, IL 60201, USA; 2 School of Medicine and Science, Rosalind Franklin University, North Chicago, IL 60064, USA; 3 Department of Neurosurgery, Endeavor Health, Evanston, IL 60201, USAjbailes@northshore.org (J.B.); 4 Department of Chemistry, Northwestern University, Evanston, IL 60208, USA; 5 The Pritzker School of Medicine, University of Chicago, Chicago, IL 60637, USA; 6 Department of Anesthesiology, Endeavor Health, Evanston, IL 60201, USA; 7 Department of Biomedical Engineering, Northwestern University, Evanston, IL 60208, USA

**Keywords:** iron oxide nanoparticles, neurological cancer, drug delivery

## Abstract

Cancers of the central nervous system are particularly difficult to treat due to a variety of factors. Surgical approaches are impeded by the skull—an issue which is compounded by the severity of possible harm that can result from damage to the parenchymal tissue. As a result, chemotherapeutic agents have been the standard of care for brain tumors. While some drugs can be effective on a case-by-case basis, there remains a critical need to improve the efficacy of chemotherapeutic agents for neurological cancers. Recently, advances in iron oxide nanoparticle research have highlighted how their unique properties could be leveraged to address the shortcomings of conventional therapeutics. Iron oxide nanoparticles combine the advantages of good biocompatibility, magnetic susceptibility, and functionalization via a range of coating techniques. Thus, iron oxide nanoparticles could be used in both the imaging of brain cancers with magnetic resonance imaging, as well as acting as trafficking vehicles across the blood–brain barrier for targeted drug delivery. Moreover, their ability to support minimally invasive therapies such as magnetic hyperthermia makes them particularly appealing for neuro-oncological applications, where precision and safety are paramount. In this review, we will outline the application of iron oxide nanoparticles in various clinical settings including imaging and drug delivery paradigms. Importantly, this review presents a novel approach of combining surface engineering and internal magnetic targeting for deep-seated brain tumors, proposing the surgical implantation of internal magnets as a next-generation strategy to overcome the limitations of external magnetic fields.

## 1. Introduction: Challenges in Neuro-Oncology

Brain tumors present significant challenges in treatment, with varying survival rates depending on the type and malignancy of the tumor. The five-year survival rate of benign tumors is 91.5%, while malignant brain and other central nervous system tumors have a five-year survival rate of 35.8%. Specifically, glioblastomas, one of the most aggressive forms of brain cancer, have a five-year survival rate of less than 10% [1,2].

Current standards of care for brain tumors typically involve a combination of surgical resection, radiotherapy, and chemotherapy. Surgery is a critical first step, aiming to remove as much of the tumor as possible, alleviate symptoms, and obtain tissue samples for optimizing the patient’s treatment plan [3]. However, the best treatment strategy for brain tumors remains debated and varies significantly depending on the healthcare facility, size, location, and the tumor’s stage at diagnosis [4].

Studies on the efficacy of different radiotherapy and chemotherapy regimens have shown mixed results. For example, Chang et al. [5] evaluated various treatments including standard-dose radiotherapy (6000 rad over 6–7 weeks), boosted radiotherapy (6000 rad over 6–7 weeks plus an additional 1000 rad over 1–2 weeks) and combinations of radiotherapy with carmustine or other chemotherapeutic agents. Their findings indicated no significant improvement in survival rates across these treatment plans. Instead, patient age was a more critical factor, with patients under 40 years showing the highest 18-month survival rate of 64%, compared to only 8% for patients over 60 years old. Additionally, treatments combining radiotherapy and chemotherapy have been associated with severe adverse side effects such as nausea, vomiting, leukopenia, thrombocytopenia, and increased intracranial pressure. Similarly, Gilbert et al. [6]. found that combining radiotherapy with escalating doses of chemotherapy did not improve survival rates for glioblastoma patients. Rather, higher chemotherapy doses led to more severe side effects due to drug toxicity. Moreover, increasing the number of chemotherapy cycles did not enhance patient survival, but did significantly increase adverse side effects [7]. These findings underscore the limitations of current treatment modalities, which result in low survival rates coupled with high levels of adverse side effects. Therefore, there is a pressing need for innovative and targeted treatment approaches specifically for brain tumors that can improve patient outcomes while minimizing side effects. However, translating these novel methodologies to a clinical setting may prove to be difficult in practice.

**Figure 1:**
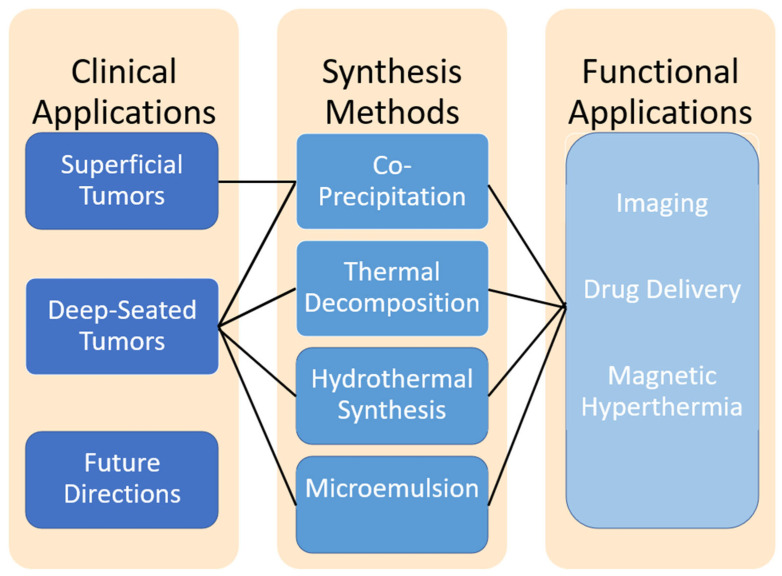
Conceptual overview of IONP synthesis methods (**center**), their key functional applications (**right**), and corresponding clinical applications (**left**). Each synthesis route—co-precipitation, thermal decomposition, hydrothermal synthesis, and microemulsion—can be tailored to produce nanoparticles optimized for imaging, drug delivery, or magnetic hyperthermia. These functionalized IONPs can then be employed to treat superficial and deep-seated tumors, with ongoing research pointing to future directions for further clinical translation.

Iron oxide nanoparticles (IONPs) exhibit unique properties such as superparamagnetism, biocompatibility, and tunable surface chemistry. These characteristics make IONPs promising candidates for various biomedical applications, including imaging and drug delivery. In the context of brain tumors, IONPs have the potential to significantly enhance treatment efficacy by enabling image-guided surgery, improving drug delivery, and facilitating localized hyperthermia. Furthermore, recent studies suggest that IONPs may play a role in modulating the tumor microenvironment and enhancing the efficacy of adjunct therapies, which is particularly relevant in the treatment of aggressive brain tumors such as glioblastoma [8]. While the clinical translation of IONPs remains a challenge, their promising preclinical advancements provide new avenues for improving treatment outcomes in neuro-oncology, offering hope for more targeted and effective therapies, outlined in Figure 1.

While prior reviews have summarized the general biomedical applications of IONPs, this review uniquely focuses on the specific challenges of neuro-oncology and introduces a novel internal magnetic targeting strategy. By proposing the surgical implantation of magnets into the tumor resection cavity, we offer a new framework to enhance IONP localization for deep brain tumors—a solution not widely explored in the existing literature. We further integrate emerging advances in personalized nanomedicine and multifunctional coatings, providing a translational roadmap for the clinical adoption of IONPs in brain cancer therapy.

## 2. Overview of Iron Oxide Nanoparticle Synthesis for Neuro-Oncology Applications

To effectively harness iron oxide nanoparticles (IONPs) for clinical applications, their synthesis must be carefully considered as the size, uniformity, and surface properties of nanoparticles significantly influence their performance in targeted therapy and imaging. Localizing the treatment to the tumor area is vital for ensuring that relevant pharmaceutical concentrations are reached. This is especially important when considering brain tumors, as the area surrounding the tumor mass should be left as intact as possible. Additionally, guaranteeing the therapeutics reach a tumor in the brain requires special attention as the drugs need to cross the blood–brain barrier (which will be discussed later). For instance, in the treatment of brain tumors, the ability to achieve precise localization—whether through external or internal magnetization—depends heavily on nanoparticle properties, which are determined by the synthesis method.

The synthesis of IONPs can be achieved through various methods, each offering different levels of complexity, versatility, and control over the uniformity of particle diameters. Given the unique challenges of brain tumor therapy, synthesis methods must prioritize precise size control, biocompatibility, and functionalization potential to ensure effective delivery across the blood–brain barrier and accumulation at the tumor site. A comprehensive summary of the various synthesis techniques can be found in Table 1 of the paper by Ling and Hyeon [9]. Here, we compare the advantages and disadvantages of four widely used synthesis methods: co-precipitation, thermal decomposition, hydrothermal synthesis, and microemulsion. We provide clinical recommendations for each method based on their suitability for superficial and deep-seated tumor treatment. To better illustrate how synthesis methods align with clinical neuro-oncology needs, we summarize key considerations in Table 1.

### 2.1. Co-Precipitation Synthesis

The co-precipitation method is commonly used in synthesizing IONPs due to its simplicity, cost-effectiveness, and the ability to produce a range of IONP sizes at room temperature or slightly elevated temperature conditions. However, limited control over size and uniformity may reduce its suitability for applications requiring precise targeting in deep brain tumors. In this method, a 2:1 molar ratio of ferrous and ferric chloride (FeCl_2_ and FeCl_3_) are combined in an alkaline environment using sodium hydroxide (NaOH) or ammonium hydroxide (NH_4_OH), which leads to the formation of ferrous (Fe(OH)_2_) and ferric hydroxide (Fe(OH)_3_), and then the precipitation of magnetite (Fe_3_O_4_) nanoparticles [10,11,12,13].

Different reaction parameters such as pH, temperature, and Fe(II)/Fe(III) molar ratio determine the size, composition, and shape of the resulting IONPs [14,15]. For example, the pH of the reaction has been reported to influence particle size formation wherein smaller particles with narrow size distribution (25–30 nm) were observed at pH ≤ 6, while a larger particle size with a broader size distribution (50–100 nm) was observed at a pH of 8 [14]. Lower pH levels tend to yield smaller nanoparticles because they promote faster nucleation and growth of the iron oxide crystals [16]. Similarly, high temperatures can also lead to smaller nanoparticles by accelerating the nucleation and growth process. It should be noted, however, that the use of higher temperatures can result in agglomeration, leading to the formation of bigger particles with a larger size distribution [16,17]. For example, Murbe et al. reported the increase in size of magnetite nanoparticles from 16 to 39 nm with increasing temperature from 25 to 90 °C [17]. Post-synthesis processing steps such as purification, size fractionation, or surface modification can be employed [18] to refine the size distribution and allow for specific applications of the IONPs.

Ultimately, the co-precipitation method is a versatile approach for the synthesis of IONPs, allowing for moderate control over size and uniformity through adjustments to the reaction parameters. This method’s use of ambient conditions and aqueous solvents is advantageous for large-scale synthesis and the commercial translation of IONPs for a wide range of applications, including magnetic resonance imaging (MRI) [19,20], targeted drug delivery [21,22,23], and hyperthermia therapy [24,25], although its clinical suitability is likely best limited to superficial brain tumors or applications not requiring high-precision delivery to the central nervous system.

Co-precipitation is the most widely used method for IONP synthesis [26,27] with the majority of commercial production using this method [28].

Clinically, we recommend the use of co-precipitation synthesis primarily for the treatment of superficial brain tumors due to following reasons:

1. Superficial tumors are more accessible to externally applied magnetic fields, meaning that extremely precise size control or advanced surface modifications may not be as critical.
2. Co-precipitation offers a cost-effective and scalable method, making it suitable for bulk nanoparticle production for superficial applications.
3. The nanoparticles can still be functionalized after synthesis for targeting or therapeutic purposes, such as hyperthermia or drug delivery.

### 2.2. Thermal Decomposition

Thermal decomposition is another versatile and widely used method for the synthesis of IONPs that offers highly precise control over their size, shape, and uniformity—critical factors when developing nanoparticles intended for crossing the blood–brain barrier and accumulating in deep-seated brain tumors. Monodispersity is highly desirable in biomedical applications to ensure consistent performance of the IONPs. Several excellent reviews have been published focusing on the design, synthesis, and formation mechanisms of IONPs with tunable morphologies and surface chemistries [9,27,29,30,31,32,33,34]. In this process, the fundamental principle involves the decomposition of organometallic precursors organic solvents with high boiling points in the presence of surfactants under an inert atmosphere. Iron (III) oleate, iron (III) acetylacetonate, or iron (0) pentacarbonyl are commonly used as precursors in thermal decomposition synthesis of IONPs [35].

The precursors are then added to solvents with high boiling points (such as benzyl ether, dioctyl ether, and 1-octadecene) in the presence of surfactants (such as oleic acid and oleylamine) via a hot injection or heating-up method [29,31,36].

Hufschmid et al. explored the use of different precursors (iron oleate, iron pentacarbonyl, and iron oxyhydroxide) using oleic acid as the surfactant while varying different parameters such as precursor/surfactant concentration, temperature, heating rate, and reaction time [37]. In their case, they found that all three precursors formed an iron–oleate complex as an intermediate and that the use of excess oleic acid led to delayed nucleation and particle growth, but also increased the particle size generated. Further, they found it necessary to perform additional oxidation of the resulting nanoparticles to generate pure magnetite or maghemite nanoparticles [37].

The influence of temperature and surfactant/ligand length and ligand concentration were recently reported by Gorke et al., where they compared the use of oleic acid/oleylamine, decanoic acid/dodecylamine, and hexanoic acid/heptylamine [38]. Their results showed that the reaction temperature, and not the ligands, controls the crystallinity and magnetization of the final product but this needs to be tailored to the boiling point of the ligand used in the reaction [38]. The thermal decomposition method forms hydrophobic IONPs, surface modifications are conducted to make the particles hydrophilic, thus improving biocompatibility, or enabling the further functionalization of the nanoparticles for specific applications [35]. Different strategies employed include coating the IONPs with gold, which is biocompatible or silica to make it hydrophilic, which can then be further modified to impart multifunctional properties to the nanoparticle (e.g., for use in imaging or drug delivery vehicle) [9]. While thermal decomposition is commonly employed at the laboratory scale, it can also be adapted for large scale production with appropriate equipment and process optimization [39]. This ease of increasing the scale of production is crucial for translating research findings into practical applications.

Thermal decomposition’s versatility and controllability make it an excellent method for synthesizing IONPs. By selecting appropriate precursors, solvents, and reaction conditions, researchers can tailor the size, uniformity, and surface properties of IONPs. This level of control is essential for customizing IONPs for various biomedical applications, and is particularly well suited for imaging [40,41].

Clinically, we recommend the use of thermal decomposition synthesis for the treatment of deep-seated brain tumors due to the following reasons:

1. Deep-seated tumors require more sophisticated and precise targeting due to the limited range of external magnetic fields and the difficulty in accessing the tumor site.
2. Thermal decomposition produces monodisperse nanoparticles with a consistent size and surface properties, critical for achieving optimal localization in challenging environments.
3. These nanoparticles are also amenable to post-synthesis functionalization for therapeutic or targeting purposes, such as hyperthermia or drug delivery.

### 2.3. Hydrothermal Synthesis

Hydrothermal synthesis of IONPs is performed in an aqueous medium under high temperature and pressure conditions. This method allows for the fine-tuning of particle size and surface properties, which is essential for optimizing nanoparticle behavior in the complex brain microenvironment. In this method, IONPs are formed by hydrolysis or iron salt precursors generating mixed metal hydroxides, which are then oxidized to form ferrite nanoparticles. Increasing the amount of water and lengthening the reaction time has been reported to lead to the formation of larger particle sizes [42,43,44,45]. The characteristics of the produced nanoparticles can be further fine-tuned by optimizing reaction parameters such as reaction time, temperature, pressure, and solution concentrations [46]. The hydrothermal synthesis method can be further improved through the use of microwave irradiation [47,48]. The addition of microwave irradiation allows for uniform thermal distribution, decreasing reaction times and increasing product yields [49]. Hydrothermal synthesis shows promise of being employed at an industrial scale as this method allows for continuous production of IONPs [42]. Additionally, hydrothermal synthesis is considered to be environmentally friendly as there is no need for organic solvents or post-synthesis treatments [50,51].

Clinically, we recommend the use of hydrothermal synthesis for the treatment of deep-seated tumors due to the following reasons:

1. Precise targeting is essential for deep-seated tumors due to their inaccessibility and the limited penetration of external magnetic fields.
2. Hydrothermal synthesis is able to produce nanoparticles with consistent size and surface properties which optimize nanoparticle localization to specific regions of interest.
3. This method has considerable means through which the nanoparticle design can be altered to suit precise applications.

### 2.4. Microemulsion Method

Microemulsion is a technique that combines oil, water, and a surfactant into a stable mixture and can be water in oil or oil in water system. While this method allows for precise size control, its limited scalability and complexity reduce its practicality for large-scale neuro-oncology applications. This method achieves IONP crystal formation through using water droplets in a solution of organic and surfactant molecules, usually bis(2-ethylhexyl) sulfosuccinate or sodium dodecyl sulfate [52]. A reducing agent is added to the solution to initiate particle formation. The reaction is then destabilized using ethanol or acetone, and is finally centrifuged to separate the IONPs [53]. Control over particle size can be achieved by varying the water to surfactant ratio or temperature used and stability of the NPs in media can be improved by the use of appropriate surfactant [54,55,56]. However, the microemulsion method has limited potential in industrial application as it is incredibly labor intensive and costly.

Clinically, we recommend the use of microemulsion synthesis for the treatment of deep-seated tumors only after careful consideration of the previously mentioned synthesis methods due to the following reasons:

1. This synthesis method allows for high control over the many facets of the nanoparticle products that allow for high specificity towards deep-seated tumor environments.
2. Microemulsion synthesis is not cost or labor effective, making scalability for industrial use difficult to justify.
3. Although microemulsion synthesis provides some control over specificity and uniformity, other methods reviewed here offer greater versatility and feasibility.

## 3. Safety and Biocompatibility in Brain Tumor Therapy

In the context of neuro-oncology, safety considerations are particularly critical given the sensitivity of brain tissue and the potential for long-term retention of nanoparticles. A vital step in cancer treatment is imaging the tumor area to determine its size and location. MRI offers clear images of brain structures and can be used in conjunction with other imaging modalities, such as positron emission tomography [57]. Successful tumor imaging through the use of MRI necessitates the use of contrasting agents to adequately distinguish between tissue types. Current contrasting agents in use for MRI are gadolinium-based products. However, findings have shown that after administration of gadolinium, a certain population of patients can develop a condition called ‘systemic fibrosis’, which is characterized by an inflammatory response that causes the thickening and eventual rigidity of the skin and joints [58]. While the etiology of this condition remains unclear, it has been reported that trace amounts of gadolinium products can remain in various tissues after administration [59,60,61]. Because there has been great concern for the safety of gadolinium-based contrast agents, copious studies have been conducted looking into the effects of gadolinium-based agents on changes in behavior and motor functioning at the cellular level. Though no definite adverse effects have been noted, remnants of gadolinium have been found months after administration in various tissues [62,63,64,65,66,67,68]. Clinical work has found that a specific patient population is susceptible to systemic fibrosis, and modifying clinical practices to minimize gadolinium use for patients with renal failure [69] has essentially eradicated this symptom. Despite this, there remains widespread concern for potential harm caused by trace levels of gadolinium remaining in patients for prolonged periods of time, leading to a safety communication from the Food and Drug Administration (FDA) [70]. Even though the data suggest that any adverse effects are avoidable, there is a lasting hesitancy regarding gadolinium use. Consequently, much research has been conducted on methods of improving the imaging ability of MR while also eliminating safety concerns, particularly for neuro-oncology applications where long-term brain tissue retention of contrast agents is of heightened concern.

IONPs have shown promising data for their use as MRI contrasting agents in brain tumor imaging [71]. Biocompatibility of IONPs have been extensively studied to ensure their safety in clinical settings, with particular attention to neural cell populations. On the cellular level, no negative effects of IONPs on cells’ morphology, proliferation, or viability have been found [72,73,74,75,76,77,78]. These studies investigate a wide range of cell populations including human neural stem cells, mesenchymal stem cells, astrocytes, microglia, neurons, and xenograft tumors. These cell types did not show any phenotype alteration when exposed to IONPs, effectively displaying the safety of using these nanoparticles for neuro-oncological applications.

In comparison to gadolinium agents, iron oxide particles are able to act as effective contrast agents for longer periods of time due to their longer half-life when in the blood stream [79,80]; for a complete list of gadolinium-based agents and their associated half-life, see the review by Ersoy et al. [81]. This property is especially valuable in brain tumor imaging, where repeated contrast administration is often required.

In addition to the studies of IONPs effects on a cellular level, there have been numerous reports that utilized animal models to investigate the biological safety of IONPs. Unterweger et al. [75] utilized a pig model to test for biocompatibility specifically in the blood through observing the complement activation-related pseudoallergy. In this study, IONPs were coated with dextran and alterations in the nanoparticles’ size were tested for their potential effects on the immune system. Upon intravenous injection, the dextran-coated nanoparticles were not only observable by MRI within 15 min of injection and through the 24 h time mark, but the nanoparticles were also shown to have no effect on the models’ immune system when observations were made to complement activation, coagulation, and hemolysis. Changes in the nanoparticles’ size did not show any effect on the results up to a 30-nanometer diameter. Through their multi-faceted study, Unterweger et al. demonstrated that, at the level of an organism, IONPs likely do not initiate an immune response, nor do they carry toxic side effects.

Upon intravenous injection of IONPs into rats, no acute or chronic side effects were observed. The nanoparticles were successfully cleared from the kidneys and bladder after a few days, and from the liver and spleen after 4 weeks [82,83]. These findings are crucial for neuro-oncology applications, where long-term nanoparticle accumulation in brain or systemic tissues could pose a safety risk.

IONP-induced toxicity has been observed on the cellular level. Multiple mechanisms of toxicity have been noted including oxidative stress [84,85,86], mutagenicity [87,88], cytoskeleton impairment [89], and iron homeostasis alteration. However, these mechanisms may be exploited for therapeutic applications, such as anti-cancer [90,91,92,93] and iron deficiency. In the context of brain tumors, controlled induction of oxidative stress by IONPs may even enhance therapeutic efficacy.

As there has been evidence of gadolinium-based products remaining in neural tissues for extended periods after their administration [61,94], concern has been raised about the potential effect of these products on an individual’s behavior. This has been addressed by the work of Habermeyer et al. [95] by monitoring rat behavior for 30 weeks after gadolinium-based product injection. No differences were found in body weight, food and water intake, emotional status, gait, or motor skills. It was, however, noted that the rats displayed a decrease in startle reactions at 7 weeks, which was recovered by 14 weeks post injection [95]. In an additional study, it has been reported that the injection of IONPs does not interfere with the memory or gross motor function of rats. Furthermore, no differences in brain tissue composition were found [96]. Many investigations have gone into the safety of IONPs, and the evidence has shown that the nanoparticles do not affect a multitude of cell populations or their basic functions. The immune system and various neural functioning of animal models are not compromised with IONP use, supporting their potential as a biocompatible platform for imaging and therapeutic applications in brain tumor treatment.

## 4. MR Imaging for Brain Tumor Visualization

Imaging neural tumors is an important step in their treatment. Locating the tumor is necessary to ensure that the tumor mass is resected in an optimal manner while leaving as much healthy tissue intact. MRI is a valuable imaging modality that provides clear and precise images. MRI is based on the magnetic properties of nuclei. Within tissue, the water nuclei are usually randomly orientated. This is disrupted by applying a pulse of radio frequency (RF) to the area and then the time needed to return to equilibrium is measured. This is defined with two relaxation times: T1 (longitudinal relaxation) and T2 (transverse relaxation). The inverse of the relaxation times, T1 and T2, are the relaxivity, noted as r1 and r2. The relaxivity ratio (r2/r1) increases with decreasing particle size, leading to better T1 imaging [97,98], making the relaxivity ratio an important indicator of effectiveness in the development of contrast agents. The T1 and T2 relaxation times, and therefore their respective relaxation rates, differ amongst tissue types, allowing for their visualization. Using contrast agents, such as gadolinium-based products or IONPs, improves the overall image obtained from MR images and can even offer specificity [99]. Effective imaging of brain tumors requires contrast agents capable of crossing the blood–brain barrier and offering high sensitivity at low doses.

Tao et al. [100] compared the effects of ‘natural’ and ‘synthetic’ macromolecules as ligand modifications in IONPs using cell toxicity, r2/r1 ratio, and the contrast produced by the particles as standards of comparison. Bovine serum albumin (BSA) was used as the ‘natural’ nanoparticle modification and poly(acrylic acid)–poly(methacrylic acid) (PAPM) as the ‘synthetic’ modification. Both types of nanoparticles demonstrated uniform diameter, similar magnetization, and low cellular toxicity. However, the BSA modification produced a high r2/r1 ratio and had a darkening contrast enhancement while the PAPM modified nanoparticle yielded a low r2/r1 value accompanied with bright contrast enhancement. The r2/r1 ratios of the ‘natural’ and ‘synthetic’ particles indicate that both are adequate T1-weighted contrast agents, with the PAPM-modified nanoparticle being the better of the two due to the lower r2/r1 value. Similarly, Wei et al. [101] produced IONPs that also demonstrated T1-weighted contrasting properties and were able to show that these particles have a higher potency per molecule compared to gadolinium-based agents [101]. This is particularly relevant in brain tumor imaging, where high sensitivity and lower required doses of contrast agents are critical.

These improvements on imaging can further ease the identification and staging of cancer by addressing the tumor’s hypoxia. Hypoxia, decreased levels of oxygen, is a hallmark of cancer that is a byproduct of a tumor’s high consumption of oxygen relative to the available oxygen from the blood. This produces molecular changes that are specific to hypoxic cells, and thus can be utilized for the imaging. By conjugating an antibody specific to hypoxia to IONPs, intense enhancement can be identified during T1-weighted imaging [102]. Such hypoxia-targeted imaging strategies are particularly promising for glioblastoma and other aggressive brain tumors known for hypoxic cores.

An alternative method for determining the hypoxic condition is through the measurement and monitoring of microvasculature in the tumor environment. IONPs can be administered and used to observe changes in the microvasculature with MRI [103]. Given the highly vascularized nature of many brain tumors, this approach offers valuable functional imaging capability.

In neuro-oncology, the ability to visualize tumor margins and residual disease is critical for surgical planning and postoperative monitoring. Intraoperative MRI is increasingly used during brain tumor resections, and IONPs hold promise as contrast agents to enhance tumor delineation in this setting. Furthermore, agents like ferumoxytol have been explored in clinical studies as off-label MRI contrast agents for brain tumor imaging, offering prolonged blood pool imaging and improved detection of tumor-associated vasculature [104].

The use of nanoparticles as T2-weighted contrast agents has also been well explored. Hachani et al. [76] tagged human mesenchymal stem cells (hMSCs) with nanoparticles and noted significant contrast enhancement in T2-weighted MRI. Through this, the proliferation and migration of implanted hMSCs was able to be noninvasively monitored. When the iron oxide nanoparticles are compared against commercially used gadolinium products, T2-weighted contrast is enhanced, and labeled regions maintain these intense areas of contrast for five weeks post administration [83]. When looking specifically for improving R2* values, cellular viability is not altered [65]. This is especially important for neuro-oncology, where repeated imaging sessions and long-term cell tracking are needed to monitor tumor progression or therapeutic cell migration.

Here, we see evidence that further establishes the use of IONPs as contrast agents for MR imaging. Furthermore, modifications can allow for these agents to be tumor-specific, easing both identification and staging of cancerous areas.

Tracking cell migration is an application that holds potential in various pathologies, including monitoring cancer progression. Multiple studies have been conducted to build the foundation of this application, and following the IONP trend of easy modifications, specificity has been shown to have promising applicability in targeting single cell types [105,106]. Ariza et al. [107] have shown that IONPs can be used to monitor the movement of single cells which were visible through MR imaging. In the context of brain tumors, such capability is highly valuable for monitoring invasive cell populations or therapeutic stem cells.

Moreover, angiogenesis, which is the rapid development of new blood vessels, represents an additional aspect of developing cancer that can be tracked with the use of IONPs. Through conjugating a marker of angiogenesis with iron oxide particles, it has been shown that an individual’s tumor burden can be tracked over time [108]. In addition, these particles were able to reduce the T2* relaxation times of the tumors [109]. Angiogenesis imaging is highly relevant for brain tumors, where vascular proliferation is a key feature of tumor aggressiveness and progression.

Therefore, the ability to track a single cell and monitor the progression of angiogenesis using IONPs offers high diagnostic specificity, coupled with the advantages of non-invasive measures. These imaging strategies strengthen the potential of IONPs as multifunctional agents in neuro-oncology, combining diagnosis, staging, and therapy monitoring.

## 5. Surface Functionalization and Targeted Delivery in Neuro-Oncology

Surface modifications of IONPS can enhance their performance in neuro-oncological applications. Here, we focus on two key aspects: (1) strategies that enable these modifications to facilitate the efficient crossing of the blood–brain barrier (BBB), and (2) the role of surface functionalization in improving nanoparticle stability, targeting specificity, and controlled drug release. By addressing both targeting and BBB penetration, this section highlights how tailored surface engineering can optimize IONP-based therapies for brain tumors.

### 5.1. Enhancing BBB Penetration

As mentioned earlier, a frequent concern when administering any pharmaceutical targeting brain tissue is the BBB. The BBB refers to the unique properties of the vasculature of the central nervous system that regulate the movement of nutrients and waste between neural tissue and the circulation from the rest of the body. While the existence of the BBB allows for excellent protection of the central nervous system from various toxins and pathogens that could be detrimental to maintaining its homeostasis, the BBB causes diminished levels of therapeutics to reach neural cells, resulting in ineffective treatment [110,111]. Unfortunately, the administration of even higher systemic doses of chemotherapeutics for brain tumors to achieve a therapeutic concentration in the brain tissue is limited due to the greater risk and severity of adverse side effects. Hence, much research has been put into the development of methods to overcome the BBB for more efficient methods of delivering pharmaceuticals to tumorous tissues in the brain.

However, even after crossing the BBB, therapeutic agents must diffuse through densely structured brain tissue to reach tumor margins, especially in regions with reduced perfusion. Recent studies have highlighted how these diffusion constraints, particularly in large human brains, pose a significant barrier to effective drug delivery and must be considered in translational strategies [112].

IONPs have shown significant promise in their ability to cross the BBB to deliver molecules to the brain [113,114,115], and have additional appeal because of their ability to target specific cell types. Anti-CD133-conjugated particles have been shown to recognize tumor areas and enhance MRI contrast of associated tumor areas [74]. Enteshari et al. [113] have shown that simply conjugating a molecule with IONPs increases the concentration in brain tissues when compared to administering the molecule alone. This study demonstrates that the use of IONPs can increase the movement of pharmaceutical molecules across the BBB to increase concentrations in the brain. This is particularly valuable for delivering therapeutic agents directly to invasive brain tumor regions. Through the use of model BBB systems, it has been shown that decreasing the diameter of the particles and increasing the zeta potential results in more efficient crossing of the BBB [114].

### 5.2. Surface Functionalization

In the context of brain tumors, surface modification strategies must address both BBB penetration and selective tumor targeting, while ensuring compatibility with neuroanatomical structures. As previously discussed, one of the many appeals of IONPs is the ease with which they can be modified. Surface functionalization is achieved through the application of biocompatible coatings and the conjugation of targeting ligands and is critical for tailoring IONP properties for improved stability, targeting specificity, and controlled drug release. This allows for their chemical and biological properties to be fine-tuned to accomplish specific outcomes. Tagging IONPs with various biological markers can allow for the particles to interact with certain cells in a highly regulated manner, ensuring that only the desired cells are imaged or treated. This is particularly appealing when addressing neurological tumors to ensure maximum therapeutic concentration at the site of the tumor.

Shevtsov et al. [115] used a glioma cell model to show that IONPs conjugated with human epidermal growth factor (hEGF) have high uptake into C6 glioma cells while retaining low cellular toxicity. This study furthers the understanding of this specific particle through the use of a rat animal model, finding that not only do the hEGF-IONPs efficiently penetrate the BBB, but the particles are also adequately retained specifically in tumor cells. Such targeted approaches hold great promise for improving the precision of brain tumor imaging and therapy.

In a similar fashion, Salehnia et al. [116] have shown that conjugating the receptor for hEGF can function as a contrast enhancement agent for MRI that precisely detects the cancer cells. Other conjugations of IONPs to specific biomarkers, including Hsp70 and specific peptide sequences, have yielded promising results in localizing the nanoparticles to particular cell types [117,118,119]. Combining IONPs with current treatments, such as radiotherapy, can lead to increased efficacy against cancerous lesions [120].

Increased specificity of the IONPs can be further achieved by combing coatings and conjugated molecules by coating IONPs with polyethylene glycol (PEG) to improve biocompatibility and conjugating the particles with doxorubicin (DOX), a common chemotherapeutic agent. With this coating combination, a longer half-life is achieved, thus allowing DOX to induce a higher degree of DNA damage, and therefore, more efficiently pushing cancer cells towards apoptosis [121]. Such approaches are particularly suited for neuro-oncology, where prolonged circulation and targeted delivery to brain tumors are essential.

Through the use of a murine model, silica-coated IONPs were conjugated to temozolomide and localized using a 0.7 T magnet. The magnet was held in place externally for only 30 min for 5 days, and the nanoparticles were observed to be significantly localized in close proximity to the magnet. It was also observed that there was a significant decrease in tumor growth and significant increase in overall survival [122]. These findings demonstrate the feasibility of magnetic targeting strategies for enhancing drug accumulation in brain tumors.

The principle of conjugating a chemotherapeutic to nanoparticles for longer retention, increased specificity towards cancer cells, and an overall better pharmaceutical profile applies to multiple chemotherapy agents [123]. By combining targeted conjugation with effective surface coatings, IONPs can be engineered not only to deliver therapeutic agents directly to brain tumor cells, but also to enhance MRI contrast, thereby improving imaging quality and ultimately surgical outcomes. Improving image quality is an important consideration for the treatment of brain tumors.

It should be noted that the coating and conjugation applied to nanoparticles can not only affect their pharmacokinetics, but also their biodistribution and clearance from the organism [124,125,126]. Certain bound proteins (IgG, fibronectin, and complement factors) can promote cell uptake through macrophage interaction [127], resulting in the accumulation in the liver and spleen [128,129]. It has been generally observed that conjugation with PEG can prevent nanoparticle recognition from the immune system, thus allowing for longer circulation time to reach more peripheral organs and cites of tumors [129,130,131]. When administered intravenously, a higher percentage of the nanoparticles end up being taken up by cells in the liver compared to any other organ or tumor site, regardless of coating or conjugation [119,132,133].

In addition to polymer coatings, gold-coated iron oxide nanoparticles have been developed to enhance biocompatibility and stability. Gold shells can improve circulation time and enable further functionalization for imaging or therapy applications [120]. Such designs have demonstrated potential in enhancing therapeutic outcomes while mitigating rapid clearance by the reticuloendothelial system. Optimizing surface modifications to reduce off-target accumulation remains a critical challenge for successful application in neuro-oncology.

## 6. Magnetic Hyperthermia in Brain Tumor Treatment

Magnetic hyperthermia therapy (MHT) is a cancer treatment that usually works in conjunction with radiotherapy or chemotherapy [134]. MHT involves injecting IONPs to the tumor area to heat the tumor cells to between 43 and 47 °C, inducing apoptosis and/or necrosis [135]. This heat is induced by activating the injected IONPs through applying an external alternating magnetic field. MHT is considered noninvasive and has shown promising safety profiles in preclinical and clinical studies, which is particularly relevant for treating sensitive regions like the brain, where minimizing adverse effects is critical [136]. In neuro-oncology, MHT offers a minimally invasive strategy to selectively ablate tumor cells while preserving the surrounding healthy brain tissue, which is especially valuable in regions where surgical resection is limited or poses a high risk.

The specific absorption rate (SAR) is a critical parameter in MHT, representing the amount of heat generated per unit mass of nanoparticles when exposed to an alternating magnetic field. High SAR values are essential for clinical applications, as they enable effective tumor heating while minimizing the required nanoparticle dose—an important consideration for reducing toxicity, particularly in brain tumor therapy where the blood–brain barrier and surrounding healthy tissue impose strict safety constraints.

One major challenge limiting the clinical translation of IONPs for hyperthermia is their tendency to aggregate in biological environments. Such aggregation leads to strong magneto–dipole interactions between particles, resulting in a significant reduction in SAR [137,138]. To address this issue, several optimization strategies have been developed, including precise control over nanoparticle size [139,140,141], morphology [141,142,143,144], and composition [145,146]. It has been shown that cube-shaped nanoparticles have a higher SAR than spherical nanoparticles [147,148].

Furthermore, doping IONPs with other ferromagnetic elements, such as cobalt or zinc has been demonstrated to substantially improve SAR and overall heating efficiency [149,150]. For example, cobalt-doped iron oxide nanoparticles exhibit enhanced magnetic properties, leading to improved hyperthermia performance [150]. Recent studies also explore hybrid nanoparticles and core–shell structures as promising designs to further increase SAR while maintaining biocompatibility—an essential requirement for safe use in neuro-oncology applications [145,146]. These advancements are critical for brain tumor therapy, where achieving therapeutic heating without damaging adjacent healthy brain tissue remains a major challenge.

MHT has been widely investigated in both animal models and clinical trials. Yang et al. [151] utilized a rabbit model to explore how MHT works in a larger animal model, using an induced tumor model and PLGA and cisplatin-conjugated magnetite IONPs. This study shows efficient localization of the nanoparticle–chemotherapeutic conjugation and effectively demonstrates the feasibility of this process on larger subjects with deeper tumors. The rabbit model’s relatively large brain size, well-developed vasculature, and ability to tolerate intracranial procedures make it a valuable translational model for testing neuro-oncology applications, including targeted hyperthermia for deep-seated brain tumors.

A meta-study conducted by Shirvalilou et al. [152] concluded that MHT is an especially effective treatment for primary glioblastoma tumors; however, they found that this treatment method showed no improvement for recurrent tumors in comparison to radio- and chemotherapies alone [152]. These findings highlight the potential of MHT as part of first-line treatment for aggressive brain tumors, though further optimization is needed for recurrent cases.

It should be noted that doping IONPs with other elements may increase their potency in MHT. The use of cobalt ferrite nanoparticles for MHT were shown to effectively eliminate cancerous cells from the tumor region [150]. Such enhanced particles could offer improved efficacy in treating glioblastoma, known for its resistance to conventional therapies.

There has been emerging evidence suggesting that a synergetic relationship exists between radiotherapy, chemotherapy, and MHT through exasperating deficiencies in DNA repair mechanisms [153,154]. This synergism holds particular promise in neuro-oncology, where combination therapies may overcome treatment resistance and improve patient outcomes. More than one modality of treatment decreases the likelihood of resistance development.

## 7. Discussion and Future Directions

IONPs represent a rapidly emerging alternative in neuro-oncology, offering significant potential for localized cancer treatment. Clinical applications compose the next major field of study for IONPs, and are slowly starting to emerge globally. Clinical trials of IONPs application include studies on breast cancer (Roger Olofsson Baggem, Sahlgrenska University Hospital, Sweden) [155], hepatic cancer (Alexander Kirichenko, Allegheny Singer Research Institute) [156], and one on glioblastoma multiform (Slawomir Michalak, Poznan University of Medical Sciences), highlighting growing interest in neuro-oncology translation.

Multiple forms of IONPs are currently approved for clinical use in the European Union including Ferucarbotran and Sienna+ [157,158,159,160,161,162,163,164,165]. An even higher number are approved in both the European Union and the United States, including ferumoxide, ferumoxsil, and ferristene [28,166,167,168]. These forms of IONPs are in used in a wide range of applications including treating iron deficiency, imaging various areas of the body, and molecular separation. Even though ample clinical opportunity has existed for the use of IONPs, approved formulations (ferumoxsil, ferristene, ferucarbotran, and ferumoxide) have been removed from the market and are used in only highly specific situations [28,167,169,170]. The NanoTherm formulation from Magforce has been developed and approved for treating tumors though MHT, marking a major milestone for IONPs in neuro-oncology [171,172]. NanoTherm has shown promise for the treatment of recurrent glioblastoma by eliciting necrosis and an immune response against the tumor cells [173].

The major limitation of current therapies lies in the lack of targeted delivery. Systemic treatments inadvertently affect healthy tissues, compromising efficacy and increasing adverse effects. IONPs provide a promising alternative, allowing for more targeted therapeutic approaches using external magnets to guide nanoparticles to the desired region.

IONPs’ greatest strength of offering high specificity may also contribute to their limited clinical use. Their role as precision tools for both imaging and therapy, especially in complex neuro-oncological settings, may limit profitability for pharmaceutical companies compared to broad-spectrum therapeutics. There is also no guarantee that clinicians would utilize such novel modalities without clear clinical workflow integration.

**Figure 2:**
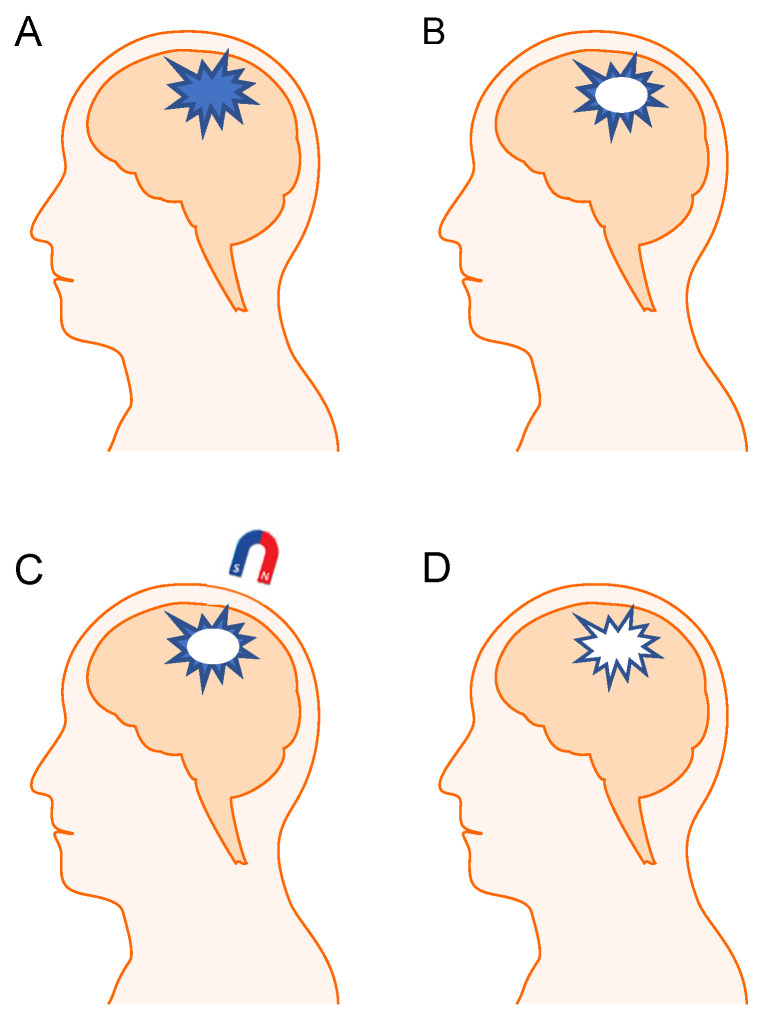
Conceptual design. Superficial brain tumor, treatable through external magnetization. Initial tumor mass shown in (**A**). Partial removal of the tumor through surgery, leaving residual cancerous cells due to the tumor’s irregular shape (**B**). After injecting IONPs, an external magnet is applied to attract nanoparticles to the tumor resection site (**C**). The localized IONPs then target residual cancer cells and potentially improve patient outcomes (**D**). Here, we recommend using IONPs synthetized with the co-precipitation method due to the simplicity and scalability of co-precipitation, which enables the rapid production of nanoparticles used in treatments involving external magnetic targeting.

External magnet-based targeting has been investigated for its potential to localize IONPs to specific regions within the body. Studies comparing tissue samples directly under the magnet with surrounding tissues have shown significantly higher concentrations of nanoparticles in the magnetized region [174,175]. Following successful localization in murine models, Patel et al. [122] designed a scalable model for human use. This model utilized 1.1 T magnet and a closed fluidics system, demonstrating that nanoparticles could be confined within a 5 cm radius of the magnet’s placement. Such approaches show promise for treating superficial tumors or accessible brain regions near the skull (Figure 2). However, the major limitations of external magnets for brain tumors are their reduced efficacy in targeting deep-seated tumors, where the magnetic field weakens, limiting nanoparticle accumulation. Passive diffusion is largely insufficient for delivering therapeutics deep into brain tissue, emphasizing the need for improved magnetic field designs or alternative targeting mechanisms for glioblastoma and other aggressive brain tumors [176].

**Figure 3:**
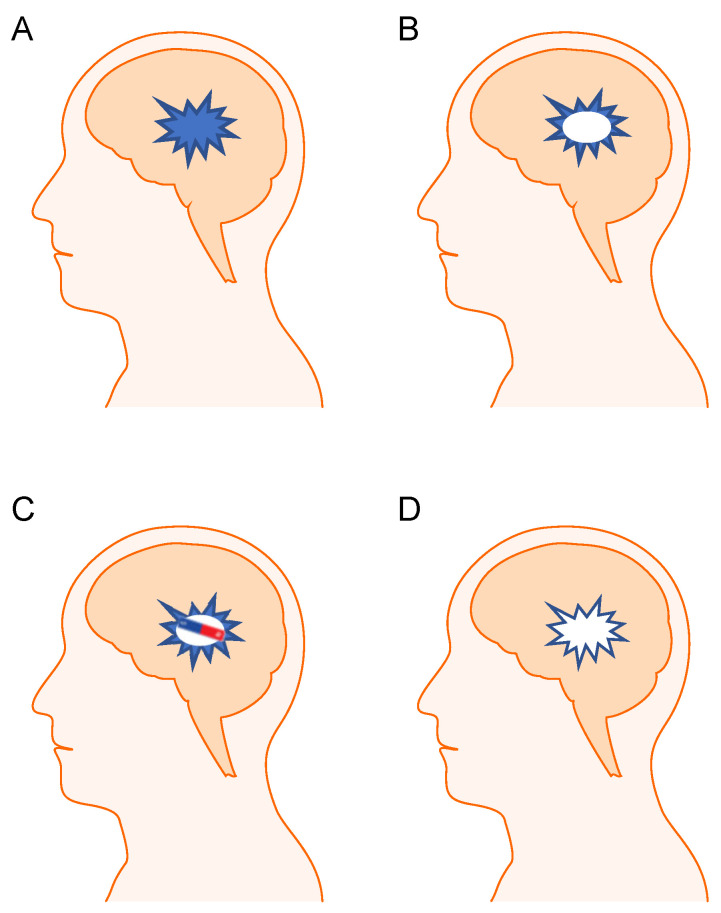
Conceptual design. The proposed treatment for a deep tumor utilizing internal magnetization. Initial tumor mass (**A**). Partial removal of the tumor during surgery, leaving some residual cancerous tissue (**B**). Placement of a magnet in the tumor cavity during surgery (**C**). Following IONP injection, the internal magnet attracts the nanoparticles to the resection site, providing stable and continuous localization. This increases the likelihood of eradicating residual tumor cells and improving patient outcomes. (**D**) Here, we recommend using IONPs synthetized with thermal decomposition method due to the precision and monodispersity which thermal decomposition provides to optimize nanoparticle localization and retention in deep-seated tumor cavities.

Despite this success with superficial tumors, deep-seated tumors remain challenging to treat due to the limited reach of external magnets. To address this challenge, we propose an innovative solution: implanting a magnet directly into the tumor resection cavity during surgery. This approach is particularly feasible in neuro-oncology, where tumor resection is commonly performed in glioblastoma and other malignant brain tumors, creating a natural space for magnet placement. This technique, displayed in Figure 3, could significantly increase the IONP concentration at the desired site without substantially extending the surgical procedure. Such a strategy has shown promise in other fields, including using magnetic stents to attract therapeutic nanoparticles [177,178,179,180,181]. Importantly, the implanted magnet could serve as a long-term targeting aid, enabling repeated IONP-based imaging or therapy sessions post-surgery—a critical advantage in managing aggressive brain tumors prone to recurrence.

Emerging therapies such as CAR-T cells, immune checkpoint inhibitors, and focused ultrasound are also being explored for brain tumors [170]. However, these approaches face challenges related to delivery, tumor heterogeneity, and immune evasion. Compared to these, IONPs offer a unique combination of targeted delivery, diagnostic imaging, and localized therapy potential, and are particularly well-suited for integration with existing surgical and radiotherapeutic workflows in neuro-oncology.

Clinical takeaway: for deep-seated tumors, we recommend using IONPs synthesized via the thermal decomposition method. This method’s ability to produce monodisperse nanoparticles with tailored surface chemistries enhances their precision, localization, and interaction with internal magnets, optimizing their therapeutic impact while minimizing off-target effects in sensitive brain structures.

### Emerging Strategies in IONP-Based Drug Development

This review introduces novel translational strategies, such as internal magnetic targeting during surgery and personalized nanoparticle engineering, which extend beyond traditional IONP applications, offering concrete pathways to overcome current clinical barriers in brain tumor therapy. One promising approach involves enhancing targeting efficiency through innovative magnetic strategies. For example, while external magnets have traditionally been used to localize IONPs to superficial tumors, our review highlights a new concept: implanting an internal magnet within the tumor resection cavity. This internal magnet approach may significantly improve nanoparticle accumulation in deep-seated tumors, overcoming the reach limitations of external fields and providing a more consistent delivery of therapeutics directly to the tumor site. By concentrating the therapeutic payload in the resection cavity, this method effectively bypasses systemic circulation, thereby reducing off-target effects and minimizing the systemic toxicity that is often associated with conventional chemotherapy. This concept aligns with previous research demonstrating that localized delivery of chemotherapeutic agents—such as with carmustine-impregnated wafers—can extend patient survival while reducing systemic side effects [182].

Additionally, the field is moving toward the integration of personalized nanomedicine. Our review highlights recent preclinical and translational studies that demonstrate how tailoring the physicochemical properties of IONPs—such as particle size, surface charge, and ligand density—can be strategically matched to the molecular profile of an individual’s tumor. We detail how fine-tuning these parameters influences key outcomes: smaller, uniformly sized particles may improve tumor penetration; optimized surface charge enhances cellular uptake and minimizes nonspecific interactions; and controlled ligand density ensures effective receptor-mediated targeting. This integrative analysis, which synthesizes findings from emerging studies, provides a concrete framework for designing personalized IONP formulations that improve targeted drug delivery and reduce off-target effects, thereby enhancing overall therapeutic outcomes for neurological tumors.

Advances in surface modification techniques are enabling the development of multifunctional IONPs. Our review is novel in that it systematically synthesizes recent preclinical studies comparing various innovative coatings—including stimuli-responsive polymers and bioactive ligands (e.g., antibodies, peptides, and aptamers)—and their direct impact on nanoparticle performance. We provide a detailed analysis of how these coatings facilitate controlled drug release and improve selective binding to cancer cells. Importantly, our review integrates these findings into a unified framework, proposing design guidelines that enable IONPs to serve a dual function: as efficient vehicles for chemotherapeutic agents, and as contrast enhancers for real-time imaging. This not only allows clinicians to monitor treatment progress and adjust dosing regimens accordingly, but also opens up new avenues for personalized therapy by tailoring surface modifications to tumor markers and microenvironments specific to the patient’s brain tumor.

Lastly, combination therapy approaches that integrate IONPs with conventional treatments such as radiotherapy, chemotherapy, and magnetic hyperthermia are emerging as a promising strategy. In this review, we utilize recent preclinical and clinical studies to demonstrate how the multifunctional properties of IONPs can be leveraged to enhance the synergistic effects of these established treatments for neurological brain tumors. IONPs not only improve the localized delivery of chemotherapeutics and enhance imaging-guided radiotherapy, but also potentiate the efficacy of magnetic hyperthermia, all while reducing systemic toxicity.

## 8. Conclusions

While IONPs hold significant promise for cancer treatment, particularly for enhancing localized therapy, key challenges remain. Effective localization strategies for deep-seated brain tumors and understanding the long-term fate of nanoparticles post-treatment are essential areas that need further research. Scaling up animal model successes and addressing questions about nanoparticle clearance will be critical in realizing the full clinical potential of IONPs. In the context of brain tumors, these challenges are even more pronounced due to the complexity of the blood–brain barrier, the high precision required to avoid damage to healthy tissue, and the urgent clinical need for new treatment strategies in aggressive gliomas.

Ultimately, overcoming these challenges could revolutionize neuro-oncology. By enabling targeted, efficient, and less invasive therapies, IONPs offer the potential to improve surgical outcomes, enhance imaging precision, and deliver localized treatments with fewer side effects. Our proposal of internal magnet placement and the integration of personalized nanomedicine represents a novel direction that may reshape the therapeutic landscape for aggressive brain tumors. Continued research into synthesis optimization, nanoparticle behavior in vivo, and novel magnetic strategies will be pivotal in realizing this vision.

## Figures and Tables

**Table 1:** Comparison of iron oxide nanoparticle synthesis methods, scalability, and clinical feasibility for neuro-oncology applications, emphasizing their suitability for superficial or deep brain tumor therapies.

Synthesis Method	Available Alterations	Industrial Scalability	Neuro-Oncology Applications	Clinical Feasibility
Co-Precipitation	Limited size control pH Post-synthesis processing	Promising for large scale Ambient conditions Aqueous solutions	Superficial brain tumors	Yes. Scalable, simple, and low-cost. Limited precision may restrict use to applications where deep targeting is not required.
Thermal Decomposition	Precise control of size, shape, and uniformity Reaction time Temperature Heating rate	Challenging for large-scale Requires organic solvents High cost of scaling up Equipment intensive	Deep-seated tumors	Yes (conditionally). Highly controlled nanoparticles ideal for precision applications like deep brain targeting, but scale up cost is a challenge.
Hydrothermal	Moderate size control Water concentration Reaction time Pressure Increased particle uniformity and yields Microwave irradiation	Very promising for industrial-scale Continuous production Multiple opportunities for specificity during production Environmentally friendly	Deep-seated tumors	Yes. Scalable and flexible, suitable for clinical production and adaptation.
Microemulsion	Precise size control Water to surfactant ratio Temperature	Not producible at an industrial scale Complex synthesis Implied high labor costs	Limited use in neuro-oncology (conceptually applicable to deep tumors)	No. Cost and complexity make it impractical for clinical production.

## Data Availability

No new data were created or analyzed in this study. Data sharing is not applicable to this article.

## Abbreviations

The following abbreviations are used in this manuscript:

BBB	Blood–brain barrier
BSA	Bovine serum albumin
DOX	Doxorubicin
FDA	Food and Drug Administration
hEGF	Human epidermal growth factor
hMSCs	Human mesenchymal stem cells
IONPs	Iron oxide nanoparticles
MHT	Magnetic hyperthermia therapy
MRI	Magnetic resonance imaging
PAPM	Poly(acrylic acid)–poly(methacrylic acid)
PEG	Polyethylene glycol
RF	Radio frequency
